# A Cross-Reference Line Method Based Multiobjective Evolutionary Algorithm to Enhance Population Diversity

**DOI:** 10.1155/2020/7179647

**Published:** 2020-07-18

**Authors:** Ya-Nan Feng, Zhao-Hui Wang, Jia-Rong Fan, Ting Fu, Zhi-Yuan Chen

**Affiliations:** ^1^Key Laboratory of Metallurgical Equipment and Control Technology of Ministry of Education, Wuhan University of Science and Technology, 947 Heping Avenue, Wuhan 430081, China; ^2^Hubei Key Laboratory of Mechanical Transmission and Manufacturing Engineering, Wuhan University of Science and Technology, 947 Heping Avenue, Wuhan 430081, China; ^3^Department of Mechanical Engineering, Institute of Manufacturing Engineering, Tsinghua University, Beijing 100084, China

## Abstract

Multiobjective evolutionary algorithms (MOEAs) with higher population diversity have been extensively presented in literature studies and shown great potential in the approximate Pareto front (PF). Especially, in the recent development of MOEAs, the reference line method is increasingly favored due to its diversity enhancement nature and auxiliary selection mechanism based on the uniformly distributed reference line. However, the existing reference line method ignores the nadir point and consequently causes the Pareto incompatibility problem, which makes the algorithm convergence worse. To address this issue, a multiobjective evolutionary algorithm based on the adaptive cross-reference line method, called MOEA-CRL, is proposed under the framework of the indicator-based MOEAs. Based on the dominant penalty distance (DPD) indicator, the cross-reference line method can not only solve the Pareto incompatibility problem but also enhance the population diversity on the convex PF and improve the performances of MOEA-CRL for irregular PF. In addition, the MOEA-CRL adjusts the distribution of the cross-reference lines directly defined by the DPD indicator according to the contributing solutions. Therefore, the adaptation of cross-reference lines will not be affected by the population size and the uniform distribution of cross-reference lines can be maintained. The MOEA-CRL is examined and compared with other MOEAs on several benchmark problems. The experimental results show that the MOEA-CRL is superior to several advanced MOEAs, especially on the convex PF. The MOEA-CRL exhibits the flexibility in population size setting and the great versatility in various multiobjective optimization problems (MOPs) and many-objective optimization problems (MaOPs).

## 1. Introduction

Multiobjective optimization problems (MOPs), which have more than one conflicting objective to be optimized, can be defined as

(1) $$\begin{array}{c} \begin{array}{cc} \text{minimize} & F\left(x\right)=\left\{f_{1}\left(x\right),f_{2}\left(x\right),\ldots ,f_{m}\left(x\right)\right\},\\ \text{subject to} & x\in \Omega , \end{array} \end{array}$$

where *F* ⊂ *R*^*m*^ is the objective vector, *m* is the number of objectives, *R*^*m*^ is the objective space, *x* = (*x*_1_,…, *x*_*n*_)^*T*^ ∈ *R*^*n*^ is the candidate solution, and Ω=∏_*i*=1_^*n*^[*a*_*i*_, *b*_*i*_]⊆*R*^*n*^ is the *n*-dimensional feasible search space. *F* : Ω⟶*R*^*m*^ defines *m* real-valued objective functions and indicates a mapping which is from the feasible search space to the objective space. Let *x*_1_, *x*_2_ ∈ Ω come to two solutions in the feasible search space. Next, if and only if *f*_*i*_ (*x*_1_) ≤ *f*_*i*_ (*x*_2_) for each *i* = 1, 2,…, *m* and *f*_*i*_ (*x*_1_) ≠ *f*_*i*_ (*x*_2_) for ∃ *j* ∈ {1, 2,…, *m*}, this shows that *x*_1_ dominates *x*_2_. If there is no any *x* ⊂ Ω which makes *F* (*x*) dominates *F* (*x*^∗^), *x*^∗^ will be called a global Pareto-optimal solution. The number of Pareto-optimal solutions is generally more than one in a multiobjective optimization problem, and the set of the Pareto-optimal solutions is named as the Pareto-optimal set. The Pareto-optimal set reflects the geometry of the Pareto front (PF) [1].

In recent years, many multiobjective evolutionary algorithms (MOEAs) have been proposed for solving multiobjective optimization problems in various fields [2–5]. In general, MOEAs have two main tasks: (1) ensuring the convergence pressure to drive populations to PF and (2) enhancing the diversity to spread populations evenly to PF [6]. If the two tasks can be accomplished well together, the approximation of PF will be better.

Although the existing MOEAs have been proved to be effective in many practical applications [7], they still have troubles in some complex multiobjective optimizations. The imbalance of convergence and diversity are the major issues since most MOEAs are designed on the principle of “convergence first and diversity second.” For instance, SPEA2 [8] and NSGA-II [9] reach the convergence based on nondominated relations, and the niche technology is used to decrease the crowding population for higher diversity. Meanwhile, the MOEAs based on decomposition such as MOEA/D [10] also choose the nondominated solutions firstly, instead of protecting the solutions with higher diversity in priority.

The imbalance of convergence and diversity makes it difficult to provide searches on different levels in the objective space. Most parts of the objective space are easier to be searched than the rest. If the nondominated solutions are not uniformly distributed, the candidate solutions may be remained far away from each other and the population may be in danger of losing diversity. In particular, it is difficult to generate feasible solutions in the untapped objective space by genetic factors since the remote parents cannot generate good offspring solutions efficiently in multiobjective optimization [11, 12]. In fact, some dominant feasible solutions can enhance the population diversity and use them appropriately can increase the pressure of choice in high-dimensional MOPs. In this sense, the diversity is as important as the convergence and should be emphasized in multiobjective optimization. Therefore, the studies on the method of diversity enhancement have been conducted to ensure a good approximation of PF. Some methods of diversity enhancement are used in the following three main types of EMO algorithms.

The first type of MOEAs is established on the fundamentals of Pareto dominance theory. The MOEAs selecting the population of next generation by Pareto dominance theory prefer nondominated individuals. The Pareto dominance theory itself does not promote the preservation of individuals with the diversity in the objective space, so the crowded strategy, niche theory, and other auxiliary strategies for diversity enhancement are proposed in order to gain the expansion of the objective space and enhance diversity. In NSGA-II, the diversity is improved by the crowded distance [9], and the crowded strategy has been extended to multiple strategies [13]. Corne et al. [14] proposed PESA-II based on regional selection strategies to improve diversity. Horn et al. [15] proposed NPGA based on dynamic niche strategy to enhance diversity.

The second type of MOEAs is built on the concept of decomposition. In MOEA/D [16], each candidate solution is linked with a subproblem and every subproblem is optimized by the information from its neighbors. Most of the decomposition-based MOEAs enhance the diversity by a uniform distribution of weight. There are two main forms of decomposition-based MOEAs. One is to decompose the initial MOPs into a series of single-objective optimization problems (SOPs), such as MOGLS [17], CMOGA [18], MSOPS-II [19], MOEA/D [16], and RVEA [20]. The other is to transform the initial MOPs into multiple more simplified MOPs through the way of dividing the objective space into multiple subspaces, such as MOEA/D-M2M [21], IM-MOEA [10], NSGA-III [11], and SPEA/R [22]. It is worth noting that the uniformity of weight distribution can enhance the diversity when a uniformly distributed weighting strategy is adopted, but the strategy may be failed in some special PFs. Therefore, the method of adaptive weights have been proposed in [23] to improve diversity in some special PFs. The method of adaptive weights have also been used in NSGA-III [11] to enhance the diversity.

The third category is known as the indicator-based MOEAs. The indicator-based theory guides the selection process by integrating the convergence and diversity into a single indicator. The advantage comparison method proposed by Sun et al. [24] enhances the diversity of evolutionary algorithms based on an inverted generational distance (IGD) indicator with the rank value and selection mechanism. Liu et al. [25] proposed a comparison algorithm that enhances the diversity of evolutionary algorithms based on the GD indicator. The comparison algorithm is not affected by the comparison order of individuals, so those solutions with good diversity can get more attention. Recently, AVREA [26] has adopted the adaptive reference line method and the achievement scalarizing function (ASF) as a/the secondary selection indicator, which efficiently enhance the diversity of AVREA.

In addition, many reference line methods have been widely used to enhance the diversity of MOEAs in recent years. For instance, MOEAs based on the nondominated sorting method (NSGA-III [11]) use a group of reference lines and choose those solutions being closer to the reference lines to enhance the diversity. Yuan et al. [27] offered an MOEA based on the reference line method that adopted a diversity enhancement mechanism similar to NSGA-III by measuring the distance between the origin and the projection of the candidate solution on the reference line. Furthermore, a method (RVEA) was proposed in [20] to adaptively modify the reference vector position based on the scale of the objective function to balance the diversity and the convergence. Recently, Sun et al. [28] proposed a MOEA based on reference lines. By the reference line method, the poles located on the coordinate axis in the objective space can be detected in order to construct a hyperplane. In particular, the boundary reference line is generated by linking the origin with the reference points on the axis, which improves the distribution of candidate solutions in the objective space. The boundary reference line is combined with the internal reference line, which divides the objective space into multiple subspaces. Subsequently, the selection for candidate solutions by reference lines enhances the diversity of the proposed algorithm. Due to the nature of the reference line method for the diversity enhancement and the auxiliary selection mechanism based on the uniformly distributed reference line, the reference line method is increasingly favored by MOEAs.

In general, the reference line method is constructed by ideal points and reference points. However, the existing reference line methods seldom take into account the influence of the nadir point although the objective space of each generation is often limited between the ideal point and the nadir point. Therefore, the influence of the nadir point cannot be ignored. In addition, when dealing with the problems with convex PFs, the reference line method can cause Pareto incompatibility problems, which makes the convergence of the algorithm worse. Considering the excitations and the defects of reference line, we propose the method based on adaptive cross-reference line. Compared to the existing reference line method, the main new contributions of this work can be summarized as follows:

1. The concept of the cross-reference line is proposed, and a new MOEA called MOEA-CRL is proposed. It inherits the advantages of the ideal point reference line in convergence, adding the nadir point reference line to enhance diversity. The ideal point reference line is combined with the nadir point reference line to divide the objective space into multiple subspaces, and the unique contributing solution preserved in each subspace to ensure the uniform distribution of the Pareto solution set. Compared with the existing reference line methods, the proposed cross-reference line method performs better in terms of diversity.
2. Based on the cross-reference line, a dominant penalty distance (DPD) is proposed to solve the Pareto incompatibility problem caused by the reference line method. Compared with the existing reference line indicators, the DPD indicator combines the properties of the ideal point reference line and the nadir point reference line, which not only solves the Pareto incompatibility problem but also improves the performance of the MOEA-CRL on the convex PF.
3. The cross-reference line adaptation method is proposed to improve the performance of MOEA-CRL for irregular PFs. The cross-reference line adaptation method not only achieves the uniform distribution by uniformly sampling points on the unit hyperplane but also adaptively adjusts the distribution of the cross-reference lines according to the contributing solutions. Compared with the existing reference line adaptive method, it adjusts the distribution of the cross-reference lines according to the contributing solutions directly defined by the DPD indicator, so the adaptation of the cross-reference line can be not affected by the population size, and the uniform distribution of the cross-reference lines can be maintained.

The rest of this paper is organized as follows. In Section 2, the PBI reference line method is analyzed in detail, and the Pareto incompatibility problem is raised. In Section 3, new reference line methods and evaluation indicators are explored to avoid the Pareto problem on the premise of fully considering the complementarity between the nadir point and the ideal point. In Section 4, the details of the proposed MOEA-CRL are mainly described. The empirical results of MOEA-CRL compared with existing MOEAs are given in Section 5. In the end, the conclusions and the future work are set out in Section 6.

## 2. Related Work

### 2.1. Reference Method Based on MOEAs

**Figure 1:**
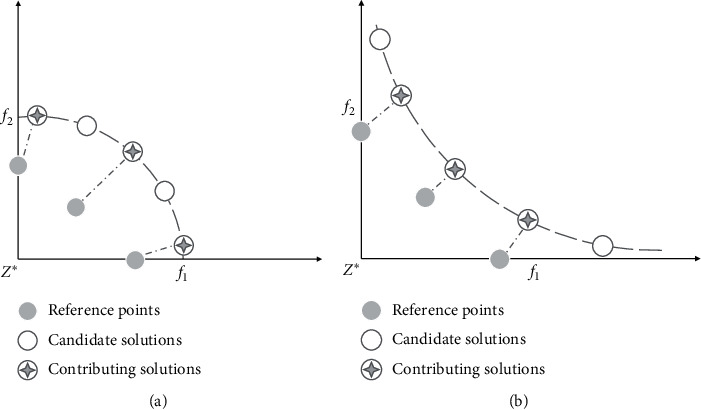
Illustration of the example containing 5 candidate solutions which are on a concave PF and a convex PF. (a) Concave PF. (b) Convex PF.

In the MOEAs based on decomposition, the existing reference line method is more advanced compared with the reference point method [29]. Especially, for the convex PF, the reference line method can effectively enhance the diversity of candidate solutions close to the coordinate axes. As the two examples given, Figure 1 shows the objective space of the two-objective optimization problems with the concave PF and the convex PF. Both examples contain five candidate solutions located on the convex PF and the concave PF, and three of them closest to the three reference points are considered as contributing solutions. If a candidate solution is closest to a reference point and the reference point is also closest to a candidate solution, the candidate solution is defined as a contributing solution of the reference point. It should be noted that, as shown in Figure 1(b), two pole-solutions close to the coordinate axis cannot be regarded as the contributing solutions.

**Figure 2:**
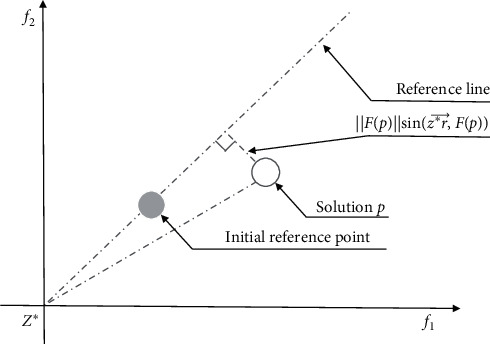
Reference line based on ideal points.

In order to improve the evaluation limitations of the reference point method, the reference point-based method has been developed into various reference line methods [29]. The distance between a candidate solution and a reference point is replaced by the distance between a candidate solution and a reference line. Therefore, a candidate solutions close to the coordinate axis can be evaluated fairly by the reference line method. As shown in Figure 2, the reference line is generated based on each reference point and the ideal point *Z*^∗^ separately. The distance between the candidate solution *p* and the reference line can be expressed as

(2) $$\begin{array}{c} \text{dis}=F\left(p\right)\sin\left(\overset{⟶}{Z^{*}r},F\left(p\right)\right), \end{array}$$

where *F*(*p*) denotes the Euclidean distance between the candidate solution and the ideal point and $\overset{⟶}{Z^{*}r}$ denotes the vector from the ideal point to the reference point.

### 2.2. PBI Reference Line Method Based on Ideal Points

The aggregation function is used as a fitness value function that weighs the merits of an individual. The aggregation function is usually a function of the individual *x* in the objective space under the condition of a given weight. The optimization of each subproblem is regarded as the optimization of the aggregation function. The PBI aggregation function is a variant of the method based on the intersection of boundaries, which aims to find the intersection point between the Pareto front and a set of lines [30]. Studies have shown that PBI aggregation functions with appropriate penalty parameter values can generate more uniform candidate solution sets, but the performance of PBI highly depends on the setting of penalty parameters that control the balance between convergence and diversity [31].

The aggregate optimization equation of the PBI function *g*_pbi_ is [32]

(3) $$\begin{array}{c} \text{Minimize}g_{\text{pbi}}\left(\left.x\right|\lambda \right)=d1+\theta \text{d}2, \end{array}$$

where

(4) $$\begin{array}{c} d_{1}=\frac{{\left(f\left(x\right)-z\right)}^{T}\lambda }{\lambda },\\ d_{2}=f\left(x\right)-\left(z-d_{1}\frac{\lambda }{\lambda }\right), \end{array}$$

where *f* (*x*) is a candidate solution, *z* is the ideal point, and *λ* is the vector of the reference line.

**Figure 3:**
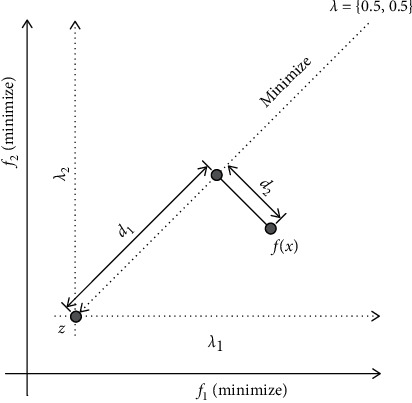
PBI reference line method [32].

PBI also uses the obtained ideal point *z* as the criterion to decompose the objective space. *θ* is the parameter of PBI, and its range is *θ* ≥ 0.  Figure 3 shows the *d*_1_ and *d*_2_ of the solution *x* of a weight vector *λ* = (0.5, 0.5)^*T*^ in the two-dimensional objective space. In the PBI method, a candidate solution with a small *d*_1_ is first considered as a better candidate solution close to the Pareto front. In addition, the distance *d*_2_ from the weight vector *λ* is considered. Finally, *g*_pbi_ is calculated by adding the value of *d*_2_ multiplied by *θ* to *d*_1_. In summary, a candidate solution with a small *d*_1_ and *d*_2_ is considered as a better candidate solution. The balance between *d*_1_ and *d*_2_ in *g*_pbi_ is controlled by the parameter *θ*. Therefore, the PBI method evolves a candidate solution toward *z* by minimizing *g*_pbi_.

### 2.3. Pareto Incompatibility

**Figure 4:**
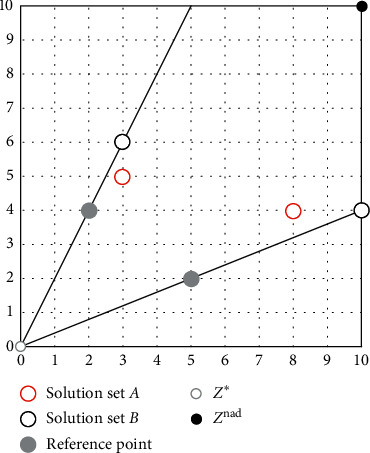
Example of Pareto incompatibility.

The Pareto incompatibility problem means that the individual's reference line evaluation results may face conflicts with the results of the nondominated relationship during the iteration process. The reference line method of the PBI aggregation function can effectively improve the diversity of candidate solutions near the coordinate axis in convex PF by increasing the value of the parameter *θ* so that the influence of *d*_2_ is much larger than the influence of *d*_1_. This method not only maintains the diversity of candidate solutions for convex PF close to the coordinate axis, but also quickly obtains the candidate solution *p* with the smallest *g*_pbi_. However, the reference line method using only ideal points causes Pareto incompatibility.  Figure 4 shows an example to illustrate the problem of Pareto incompatibility.

It can be clearly seen from Figure 4 that this simple example is a two-objective minimization problem, where the reference point set is {(2, 4), (5, 2)}, and the candidate solution set *A* = {(3, 5), (8, 4)} and the candidate solution set *B* = {(3, 6), (10, 4)}. The candidate solution set *B* = {(3, 6), (10, 4)} is the smallest distance *d*_2_ from the reference line, so the candidate solution set *B* is the best. However, according to Pareto dominance theory, (3, 5) in solution set *A* dominates (3, 6) in candidate solution set *B*, and (8, 4) in solution set *A* dominates (10, 4), so candidate solution set *A* is better than candidate solution set *B*. Therefore, the PBI aggregate function may cause the judgment of the merits of the solution set to be contrary to Pareto dominance theory.

### 2.4. Complementarity of the Nadir Point and the Ideal Point

The setting of the reference point in the aggregation function plays a key role in the performance of MOEA/D. In fact, different types of reference points may have different effects on the exploration behavior of MOEA/D. Most MOEA/D improvements use ideal points as reference points. As mentioned in [33], when diversity is easy to maintain, the method using only ideal points will be effective, and only using ideal points is more helpful to promote candidate solutions to approximate PF. In [34], MOEA/D uses ideal point and a set of reference points evenly distributed along the convex PF to ensure good population diversity. In [32, 35], MOEA/D attempts to introduce the nadir point as a reference point. In [32], the reverse PBI function is proposed, and the nadir point is used to solve the reverse PBI to maximize the value of the aggregate function, which improves the search performance of MOEA/D. In [35], if the candidate solutions obtained in the boundary area after several generations are less than the PF intermediate area, the reference point will change from the ideal point to the nadir point. Recently, Wang et al. [36] studied the effect of the difference between the ideal point and the nadir point on the performance of the algorithm and showed that they can complement each other.

**Figure 5:**
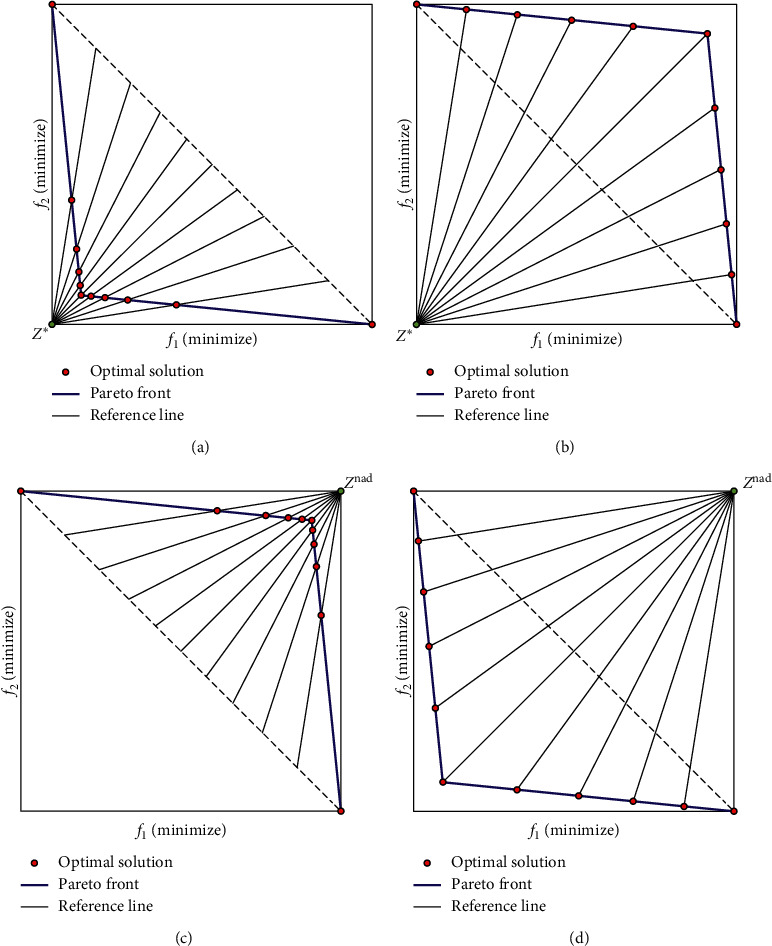
Optimal solution distribution on PF when ideal point *z*^∗^ and nadir point *z*^nad^ are used as reference points.

As Wang et al. [36] pointed out, the use of the ideal point *z*^∗^ and the nadir point *z*^nad^ in the Chebyshev function has an important influence on the distribution of the optimal solution on the PF. In particular, in the case where the ideal point *z*^∗^ is used as a reference point, the optimal solutions of the subproblems of convex PF and concave PF are shown in Figures 5(a) and 5(b), respectively. It can be clearly seen that the optimal solution density of the central part of the convex PF is much larger than that of the concave PF, but it is opposite near the PF boundary. Compared with the ideal point *z*^∗^, if the nadir point *z*^nad^ is used as a reference point, the distribution directions of the optimal solutions on these PFs are reversed, as shown in Figures 5(c) and 5(d), respectively. Since the final population distribution obtained by using the ideal point *z*^∗^ and the nadir point *z*^nad^ is complementary, using them as reference points at the same time may improve their performance, making them approximate convex PF and concave PF. In addition, if the nadir point *z*^nad^ is not used as a reference point, you may face greater diversity risks when it is not easy to maintain diversity.

## 3. The Proposed Cross-Reference Line Method

### 3.1. Cross-Reference Line Method

**Figure 6:**
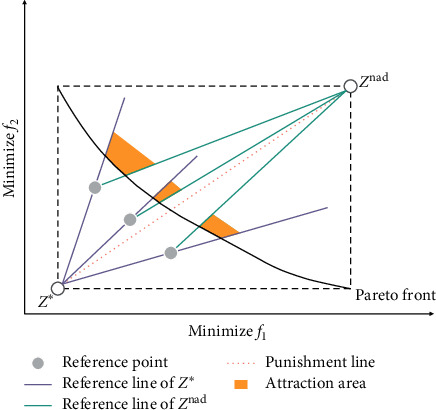
The cross-reference line method.

The cross-reference line is formed by matching the ideal point reference line and the nadir point reference line one by one. As shown in Figure 6, based on the nadir point and each reference point, the nadir point reference line is constructed, and based on the ideal point and each reference point, the ideal point reference line is constructed. The ideal point reference line and the nadir point reference line corresponding to each reference point intersect at this reference point and form an angle area in the feasible area. The boundary of the angle area is composed of an ideal point reference line and a nadir point reference line and the Pareto front edge being sandwiched. The candidate solution approaches the Pareto front under the pressure of convergence. With each generation of calculation, the candidate solution tends to move into the included angle area, which is called the attraction area.

It is worth noting that if a reference line is defined as the line between the ideal point and the nadir point, the distance between the candidate solution on the reference line and the ideal point reference line and the nadir point reference line is zero. Therefore, it will have an absolute advantage and break the fairness of candidate evaluation. In order to solve this problem, the connection between the ideal point and the nadir point is defined as the penalty line of the cross-reference line, and a certain additional penalty value is added to the candidate solutions that fall on the connection line. Therefore, the candidate solution on the penalty line can only be considered as a contributing solution to other cross-reference lines near the penalty line.

### 3.2. DPD Evaluation Indicator Based on Cross-Reference Line Method

The DPD evaluation indicator of the cross-reference line method is based on the ideal point reference line distance *d*_∗_ and the nadir point reference line distance *d*_nad_. Among them, the equation of the ideal point reference line distance *d*_∗_ and the nadir point reference line distance *d*_nad_ is as follows:

(5) $$\begin{array}{c} d_{*}=F\left(p\right)\sin\left(\overset{⟶}{Z^{*}r}|,|F|\left(p\right)\right),\\ d_{\text{nad}}=F\left(p\right)\sin\left(\overset{⟶}{Z^{\text{nad}}r}|,|F|\left(p\right)\right), \end{array}$$

where *F*(*p*) represents the Euclidean distance from the ideal point or reference point to the candidate solution *p*, $\overset{⟶}{Z^{*}r}$ represents the vector from the ideal point to the reference point, $\overset{⟶}{Z^{\text{nad}}r}$ represents the nadir reference point vector, and *F*(*p*) represents the vector from the ideal point or the nadir point to the candidate solution *p.*

Based on the cross-reference line method, the dominant penalty distance (DPD) indicator is defined as the maximum value of the ideal point reference line distance (*d*_∗_) and the nadir point reference line weighted distance (*d*_nad_). The weighting factor *μ* is used to verify the effectiveness, ensure effective enhancement of diversity, and improve the performance of MOEA-CRL. The equation of the cross-reference line DPD indicator of the nondominated candidate solution *p* is as follows:

(6) $$\begin{array}{c} {\text{DPD}}_{p}=\max\left(d_{*},\mu d_{\text{nad}}\right), \end{array}$$

where *d*_∗_ is the distance from a candidate solution to the ideal point reference line, *μ* is the weight coefficient, and *d*_nad_ is the distance from a candidate solution to the nadir point reference line.

**Figure 7:**
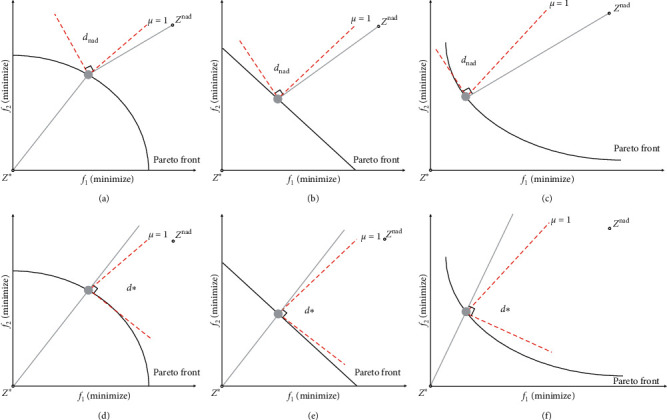
Cross-reference line DPD indicator under different PF when *μ* = 1.

Taking the weighting factor *μ* = 1 as an example, the DPD indicator is dealing with different types of PF problems, as shown in Figure 7. According to (6), the angle area between the boundary line of *μ* = 1 and the vertical line of the nadir point reference line is dominated by the distance *d*_∗_ of the nadir point reference line. The angle area between the boundary line of *μ* = 1 and the vertical line of the ideal point reference line is dominated by the ideal point reference line distance *d*_nad_.

**Figure 8:**
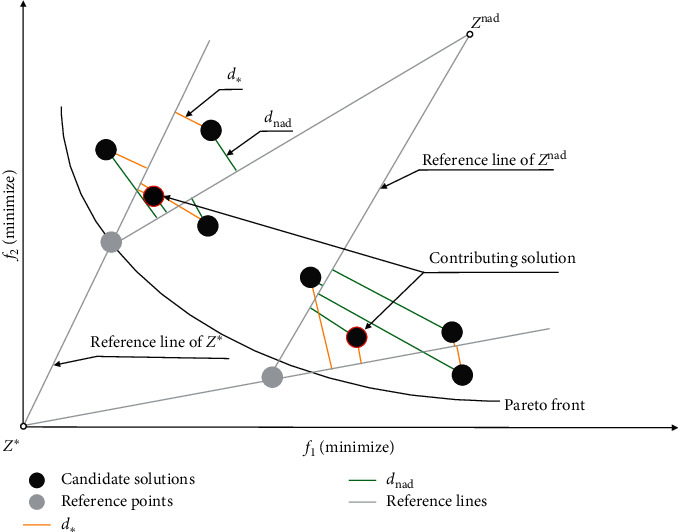
Evaluation method of the DPD indicator based on cross-reference lines.

The basic idea of the DPD indicator proposed in this paper is to combine the nadir point and the ideal point and use the cross-reference line as the evaluation reference. This method can not only effectively improve the diversity of candidate solutions near the coordinate axis in convex PF but also ensure the convergence under Pareto's dominance theory. As shown in Figure 8, the evaluation method of the DPD indicator based on cross-reference lines is shown. According to the definition of the contributing solution, the nondominated candidate solutions with the smallest DPD*p* value in Figure 8 are the contributing solutions. This idea can be regarded as the combination of the Pareto theory of advantages and the distance evaluation between the cross-reference line and the candidate solution.

### 3.3. Convergence and Diversity of the Cross-Reference Method

**Figure 9:**
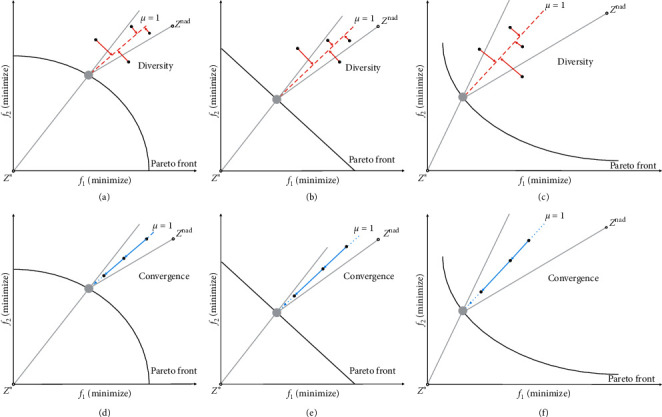
Convergence and diversity of the cross-reference line method under different PF when *μ* = 1.

The cross-reference line method enables MOEAs to ensure good convergence and diversity when dealing with various types of PF problems. As shown in Figure 9, taking weight coefficient *μ* = 1 as examples, multiple sets of examples showing the two-objective minimization problem of concave PF, convex PF, and linear PF are shown. If there is a candidate solution corresponding to a reference point with the smallest DPD value and the candidate solution corresponding to the reference point has the smallest DPD value, then this candidate solution is referred to as the reference point contributing solution. In this paper, if a candidate solution *p* has the smallest DPD_*p*_ to a cross-reference line and the cross-reference line also has the smallest DPD_*p*_ to the candidate solution *p*, the candidate solution is defined as a contributing solution of the cross-reference line. According to DPD evaluation indicator equation (6) and the uniformly distributed reference point set, each of reference points has the unique contributing solution from all candidate solutions. Therefore, the contributing solution of a reference point will tend to be close to the boundary of *μ* = 1 as the iterative search proceeds. In short, the contributing solution tends to be close to both the nadir point reference line and the ideal point reference line.

**Figure 10:**
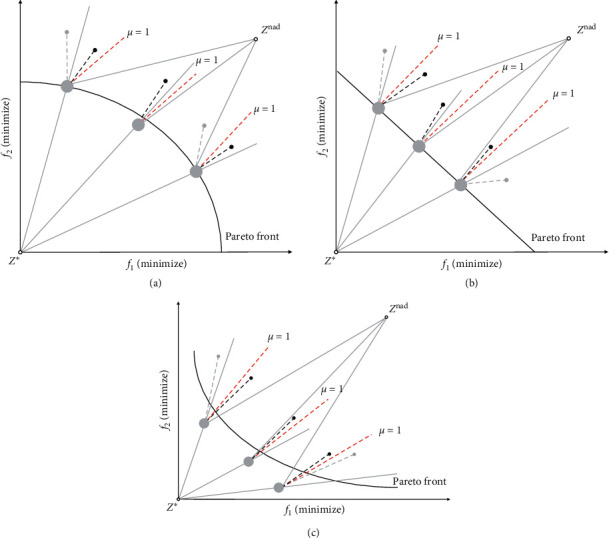
Cross-reference line method under different PF when *μ* = 1.

As shown in Figure 10, taking weight coefficient *μ* = 1 as examples, multiple sets of examples showing the two-objective minimization problem of concave PF, convex PF, and linear PF are shown. Each reference point in the reference point set matches the nadir point and the ideal point, so that the feasible search area is divided into multiple subspaces. According to DPD evaluation indicator equation (6) and the uniformly distributed reference point set, each of subspaces has unique contributing solution, thus ensuring the diversity and uniform distribution of the cross-reference line method.

The cross-reference line method is an improvement to the reference line method, inheriting the advantages of the ideal point reference line in terms of convergence and adding the nadir point reference line to enhance diversity. The cross-reference line is the combination of the ideal point reference line and the nadir point reference line to divide the objective space into multiple subspaces, and unique candidate solution with the best convergence is kept in each subspace to ensure uniform distribution of the Pareto solution set.

### 3.4. Convergence and Diversity of Cross-Reference Method

**Figure 11:**
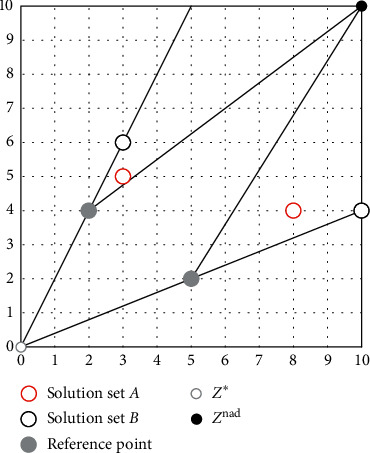
Example of the cross-reference method to avoid Pareto incompatibility.

As shown in Figure 11, the weighting factor *μ* = 1 is used as an example, and other parameters are the same as those in Figure 4. The distance *d*_∗_ of the candidate solution (3, 6) to the ideal point reference line is smaller than the distance *d*_nad_ to the nadir point reference line, so the DPD indicator of (3, 6) is dominated by *d*_nad_. The distance *d*_∗_ from the candidate solution (3, 5) to the ideal point reference line is greater than the distance *d*_nad_ from the nadir point reference line, so the DPD indicator of (3, 5) is dominated by *d*_∗_. It can be calculated that the DPD value of (3, 6) is greater than (3, 5), so (3, 5) in the candidate solution set *A* is the best. (8, 4) and (10, 4) can be compared in the same way. In summary, it can be seen that candidate solution set *A* is superior to candidate solution set *B*. According to the dominance theory, (3, 5) in solution set *A* dominates (3, 6) in candidate solution set *B*, and (8, 4) in solution set *A* dominates (10, 4) in candidate solution set *B*. Therefore, candidate solution set *A* is superior to candidate solution set *B*. Therefore, the judgment of the DPD evaluation indicator on the merits of the candidate solution set is the same as Pareto's dominance theory.

According to DPD evaluation indicator equation (6) and the uniformly distributed reference point set, each of subspaces has unique contributing solution and the contributing solutions of each subspace are jointly influenced by the ideal point reference line and the nadir point reference line. Therefore, Pareto incompatibility can be effectively avoided. The cross-reference line method not only solves the Pareto incompatibility but also can quickly obtain the nondominated candidate solution *p* by calculating the DPD_*p*_ indicator.

## 4. The Proposed Algorithm

In this section, we first describe the overall framework of the proposed MOEA-CRL in detail. Then, the case study on the implementation of the adaptive cross-reference line method is demonstrated in detail. Finally, the environment selection based on the DPD indicator is illustrated in detail, and the differences on environment selection between MOEA-CRL and other MOEAs are analyzed.

### 4.1. The General Framework of MOEA-CRL

In this section, the general framework of the MOEA-CRL will be elaborated through employing the cross-reference line method and the DPD indicator on the existing fundamentals of MOEAs. The cross-reference lines are formed by the intersection of the ideal point reference lines and the nadir point reference lines. As shown in Algorithm 1, the computing flow is divided into two steps. The first step is to initialize, and the second is to optimize.

The initialization provides preparation for MOEA-CRL. Firstly, a random initialized population is generated according to the initial parameters, which include the number of objectives *M*, the variable number *D*, the population size *N*, the maximum evolution generations, and so on. Secondly, the initial population is sorted according to the efficient nondominated sort (ENS) [37], and the nondominated solution set is copied to the initial archive *A*. Thirdly, the hyperplane is built based on initial archive *A* and the points on the hyperplane are uniformly sampled. Therefore, the uniform distribution of the initial reference point are insured.

The optimization is the core of the MOEA-CRL. The mating pool is established according to the tournament selection strategy. The DPD is used as an evaluation indicator to calculate the fitness value *fitness*_*p*_ of each candidate solution *p* to select individuals in the mating pool. The equation of distribution *fitness*_*p*_ can be expressed as

(7) $$\begin{array}{c} fitness_{p}={\text{DPD}}_{\max}-\text{DPD}\left(\frac{\left\{p\right\}}{P,R^{\prime }}\right), \end{array}$$

where DPD_max_ represents the maximum value of DPD in the population, *R*′ represents the updated reference point set, *p* represents a candidate solution, and *P* represents the total population. The establishment of the mating pool and the individual selection process based on the cross-reference line method and DPD indicator are operated in detail in Algorithm 2. It can be divided into two steps. In the first step, each individual is normalized and the *fitness*_*p*_ of each individual is calculated separately. In the second step, the individual selection is employed by the tournament selection strategy, which can randomly select two candidate solutions for comparison and then retain the individual with the larger *fitness*_*p*_. In general, the mating pool with the number of *N*/2 is obtained, and a new population *O* with the number of *N* is obtained after the mutation operation.

### 4.2. The Cross-Reference Line Adaptation

The adaptive cross-reference line method is a key step in the optimization. As shown in Algorithm 3, the method contains six operations: (1) deleting the duplicate candidate solutions (the absolute error of each objective value of the two candidate solutions is less than the given precision *ε*, which is defined as the repeated candidate solution. In the paper, the precision is *ε* = 1*e* − 6.) and dominant solutions in the archive *A*, (2) updating the ideal points and nadir points, (3) normalizing the archive *A* and the reference point *R* and calculating the DPD indicator for each candidate solution, (4) calculating the contributing solution set and effective reference points, (5) updating the archive *A*, and (6) updating the reference point *R*′ of the cross-reference lines to update the cross-reference lines.

In the second step of Algorithm 3, the update of the ideal point and the nadir point depends on the archive A for each generation, which provides the support for the normalization of different objective functions and calculation of the DPD indicator. In the third step of Algorithm 3, the archive A and the reference points are normalized to the same interval ∏_*i*=1_^*M*^[0, *z*_*i*_^nad^ − *z*_*i*_^*∗*^], so the influence of the difference objective functions is eliminated, which is convenient to compare. In addition, the calculation of the reference line distance according to the DPD indicator is shown in Algorithm 4, and the specific equation is shown (6).

In the fourth step of Algorithm 3, the contributing solutions will be calculated, and finally the contributing solution set is obtained. The contributing solution must satisfy that the solution *p* has the smallest DPD_*p*_ for a cross-reference line. Through the calculation of the DPD evaluation indicator, all contributing solutions are copied from *A*^con^ to the new archive *A*′, and the remaining space of *A*′ is filled up by candidate solutions from *A*\*A*′ one by one until *A*′ reaches its maximal size of min (|*R*|, |*A*|), where at each time, the candidate solution *p* having the maximum value of min_*p1∈A\A*;*p2∈A*′_ arccos (*f* (*p1*), *f* (*p2*)) in *A*\*A*′ is copied to *A*′, with arccos (*f* (*p1*), *f* (*p2*)) indicating the acute angle between *p*1 and *p*2 in objective space. In this way, the archive always contains a number of nondominated solutions with good distribution. It is worth noting that these nondominated solutions with good distribution, which are noncontributing and selected into *A*′, will serve as supplement for subsequent reference point updates.

The fifth and sixth steps of Algorithm 3 are the key of the cross-reference line adaptation method. Firstly, the valid reference point set *R*^*valid*^ is obtained. The valid reference points must satisfy two conditions at the same time: (1) the solution *p* has the smallest DPD_*p*_ for the cross-reference line and (2) the cross-reference line with the solution *p* has the smallest DPD_*p*_. The calculation of the DPD indicator will be affected by the distance between the candidate solution *p* to the ideal point reference line and the nadir point reference line at the same time and is calculated according to (6). Subsequently, the valid reference point set *R*^*valid*^ is copied into the reference point set *R*′. Finally, the remainder of *R*′ is complemented by the candidate solutions in the new archive *A*′ until |*R*′| = min (|*R*|, |*A*′|) is satisfied. The complementary strategy is to calculate the maximum value of min_*r∈R*′_ arccos (*f* (*p*), *r*), and the remaining part of *R*′ is the candidate solutions in *A*′ corresponding to these maximum values.

**Figure 12:**
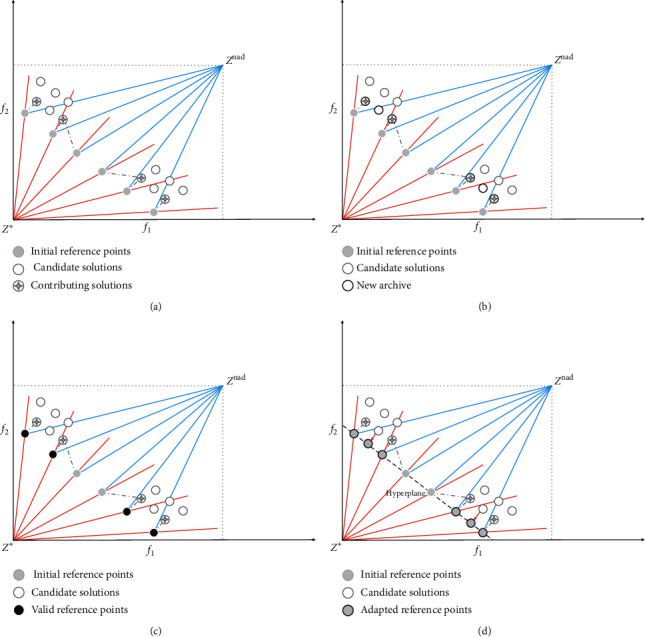
Illustration of the process of reference point adaptation. (a) Based on the DPD indicator, the four candidate solutions all meet the conditions of the contributing solution. (b) The four contributing solutions and two noncontributing solutions are copied to the new archive *A*′. (c) The four reference points satisfying the conditions of the valid reference point are copied into the valid reference point set *R*^*valid*^. (d) The four valid reference points from *R*^*valid*^ and the projection points of the two candidate solutions in the new file *A*′ on the hyperplane are copied to the set of reference points *R*′.

Figure 12 shows the adaptive update process of the reference point set *R* and the archive *A*. Firstly, four contributing solutions are obtained by calculating the DPD indicator of the candidate solutions to each reference point as indicated in Figure 12(a). Secondly, the four contributing solutions and the other two noncontributing solutions are copied into the new archive *A*′ as illustrated in Figure 12(b), where the two noncontributing solutions are nondominated solutions with good distribution and elected into *A*′ according to the maximum value of min_*p1∈A\A*; *p2∈A*′_ arccos (*f* (*p1*), *f* (*p2*)) in *A*\*A*′. Thirdly, the cross-reference lines which the contributing solution have the smallest DPD for are copied into the valid reference point set *R*^*valid*^ as shown in Figure 12(c). Finally, the projection points of the two candidate solutions in the new file *A*′ on the hyperplane and four valid reference points are copied into *R*′ as shown in Figure 12(d).

The adaptation of reference points not only ensure their own uniformity but also reflect the geometric property of the PF. Therefore, after updating the reference points, the cross-reference lines can also adaptively update to improve the performances of MOEA-CRL for irregular PF.

### 4.3. Environmental Selection Based on DPD Indicator

The environment selection based on the DPD indicator presents as shown in Algorithm 5. Being similar to most MOEAs, MOEA-CRL uses an elite strategy to make environmental choices for each generation. It is worth noting that after normalization and ENS, the smallest *k*-th generation whose individual number reaches N needs to be selected to enhance diversity, and the DPD indicator is employed for the selection.

Although the selection of most decomposition-based evolutionary algorithms is guided by a set of reference points, the reference lines in MOEA-CRL have different purposes. In the MOEA-CRL, the cross-reference lines are adopted to calculate the DPD indicator to evaluate candidate solutions, but each candidate solution is associated with unique reference point in the decomposition-based MOEAs. Therefore, the population size of MOEA-CRL can be unequal to the number of reference points and is not necessarily the same as the method proposed by Das and Dennis [37].

In addition, MOEA-CRL adjusts the distribution of the cross-reference lines according to the contributing solutions directly defined by the DPD indicator, so the adaptation of the cross-reference line can be not affected by population size, and the uniform distribution of cross-reference lines can be maintained. Regardless of the size of the population, MOEA-CRL is always able to obtain uniformly distributed candidate solutions, providing the flexibility for population size settings. This conclusion is further evidenced by the empirical results in Section 5.4.

## 5. Experimental Results and Analysis

In this section, the sensitivity analysis of the DPD weight coefficients is firstly conducted. It not only proves the validity of the cross-reference line method but also offers the best weight coefficient *μ* of the MOEA-CRL. Subsequently, the parameters are set in detail. In Section 5.3, the proposed MOEA-CRL is compared with four existing popular MOEAs, including MOEA/D [16], NSGA-III [11], RVEA [20], and KnEA [38]. Finally, the sensitivity analysis of the population size of the MOEA-CRL was performed.

In the experiment, 19 test problems from three widely used test suites, including DTLZ1-DTLZ7 [39], WFG1-WFG9 [40], MaF3, MaF11, and MaF15 [41], were used to verify the algorithm in this paper. DTLZ1-DTLZ7 and WFG1-WFG9 are the problems of the quantity of scalable objectives, which are used to test the performances of the MOEAs on various MOPs and MaOPs. MaF3, MaF11, and MaF15 possess highly irregular “convex” PFs which can be used to test the performances of algorithms on the highly irregular “convex” PF.

### 5.1. Sensitivity Analysis of Weight Coefficients of MOEA-CRL

In the MOEA-CRL, the maximum of the ideal point reference line and the nadir point reference line is selected as the DPD indicator, so the choice of the DPD weight coefficient *μ* significantly affects the performance of the MOEA-CRL. The ideal point reference line determines the convergence performance of the MOEA-CRL, and the supplement of the nadir point reference line not only solves the Pareto incompatibility but also enhances diversity. The weighting coefficient *μ* changes the fairness of the ideal point reference line distance and the nadir point reference line penalty distance, which will affect the convergence and population diversity of MOEA-CRL. Therefore, the weight coefficient *μ* is an important factor that determines the performance of MOEA-CRL. A suitable *μ* is set to meet requirements of the convergence and diversity, which helps to enhance the flexibility of the MOEA-CRL for different MOPs and MaOPs.

**Figure 13:**
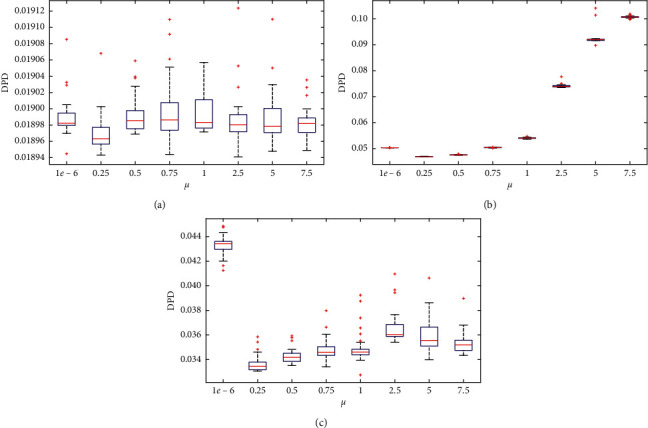
Sensitivity tests for DPD weight coefficient *μ*.

In this section, in order to study the effect of the weight coefficient *μ* on the performance of MOEA-CRL, the different *μ* values are employed for performance comparison. In the experiment, *μ* was set to 1*e* − 6, 0.25, 0.5, 0.75, 1, 2.5, 5, and 7.5, respectively. *μ* was set to 1*e* − 6 to prove the role of the nadir point reference line and further prove the effectiveness of the cross-reference line method. In order to research the effect of the weight coefficient *μ* on the proposed MOEA-CRL, the test problems select three kinds of test problems “linear,” “convex,” and “concave” according to the feature of the PF, such as DTLZ1, DTLZ2, and MaF3. The DTLZ1, DTLZ2, and MaF3 were tested with 3 objectives. In addition, the other parameter settings of MOEA-CRL are the same as in Section 5.2. A box-plot of the DPD indicator obtained for the eight *μ* cases among the three test problems is indicated in Figure 13.

It can be seen from Figure 13 that the mean DPD with the weight coefficient *μ* = 1*e* − 6 is larger than the mean DPD with *μ* = 0.25. The results show that the complement of the nadir point reference line even can increase the convergence pressure, which can improve the convergence performance of MOEA-CRL, in the case of a specific weight coefficient *μ*. It can be seen from Figure 13(a) that the mean DPD does not change significantly as the weight coefficient *μ* changes, which can be found that the weight coefficient *μ* has little influence for linear PF. As illustrated in Figure 13(b), the mean DPD changes drastically. When the weight coefficient *μ* > 0.25, the mean DPD increases as *μ* increases. It is particularly noteworthy that when *μ* > 1, the mean DPD increases dramatically. This indicates that when the weighting coefficient *μ* is so large that the convergence performance of the MOEA-CRL is significantly deteriorates for concave PF. The reason is that the population convergence pressure will gradually decrease for concave PF as the weight coefficient *μ* increases. It is shown in Figure 13(c) that the mean DPD with the weight coefficient *μ* = 1*e* − 6 is the biggest. This indicates that the complement of the nadir point reference line can enhance convergence for convex PF. It proves that it is valid to employ the nadir point reference line in the cross-reference line method as a strategy for evaluation. Furthermore, Figure 13(c) illustrates that the convergence performance of MOEA-CRL with the weight coefficient *μ* = 0.25 is optimal compared with other cases. Therefore, in the work of this paper, the weight coefficient *μ* is set to 0.25.

### 5.2. Experimental Settings

In order to compare fairly with existing advanced algorithms, this article uses general parameter settings, as follows:

1. Setting of the reference point. The reference point generation of MOEA/D, NSGA-III, and RVEA is on the basis of the two-layer method proposed by Das and Dennis [37].  Table 1 makes a list of the number of reference points in the test experiment for each objective quantity, in which *p*1 and *p*2 represent the number of divisions of each objective of the boundary layer and the inner layer, respectively. For fair comparison, MOEA-CRL also uses the same number of preset reference points listed in Table 2, and the population size of all MOEAs is the same as the number of reference points.
2. Relevant parameter settings of the competition algorithm. In MOEA/D, the size of neighborhood *T* is set to 1/10 of the population size, and the aggregate function used by the algorithm is the Chebyshev method. The penalty parameter *α* of RVEA is set to 2, and the reference point adaptive frequency *fr* is set to 0.1. The preset parameter *T* of KnEA is 0.5. There are no additional parameters for NSGA-III.
3. Genetic operation. The crossover operators in all experiments in this experiment are analog binary crossovers, and the mutation operators are polynomial mutations [42]. The distribution indicators of the crossover operators and the mutation operators are both set to 20, and the crossover probability and the mutation probability are set to 1.0 and 1/*D*, respectively, in which *D* represents the number of decision variables.
4. Performance indicators. The convergence and the diversity of the solution sets are indicated by the IGD and the hyper-volume (HV). In the HV calculation, all individuals of the population have been normalized, then the normalized HV value is calculated with a reference point (1.1, 1.1,…, 1.1). The MOEA with a larger HV value has better performance than the other. In addition, in order to reduce the computational complexity and improve the computational efficiency, the Monte Carlo estimation method is adopted for problems with the objective number is 5 and 10, and the number of sampling points required for the calculation is set to 1,000,000. In the DPD calculation, approximately 5,000 uniformly distributed points are sampled at the PF by the two-layer method proposed by Das and Dennis [37]. All tests were run 30 times independently, and the mean and standard deviation of each metric were recorded. The results of the experiment were statistically analyzed by the Wilcoxon rank sum test with a significance level of 5%, as Tables 3 and 4, where the symbol “+” indicates that the result of the other MOEA is significantly better, the “−” indicates that the result of the other MOEA is significantly worse, and “≈” indicates the similar performance of MOEA-CRL.

### 5.3. Comparisons between MOEA-CRL and Existing MOEAs

Table 3 lists the comparison of the mean values of the IGD results on the test problems with 3 objectives between MOEA-CRL and four popular MOEAs. It can be seen from the evaluation results of the mean value of the IGD in Table 3 that the MOEA-CRL proposed in this paper is superior to the other four MOEAs in dealing with test problems for 3 objectives. Among 10 test problems with regular PFs, 9 test problems except DTLZ4 obtained the best solutions by MOEA-CRL. The mean value of the IGD of MOEA-CRL was slightly larger than RVEA on DTLZ1 and NSGA-III on WFG6. The MOEA-CRL also shows the good performance on the test problems with 9 irregular PFs. Especially, on the MaF3, MaF11, and MaF15 problems with three concave PF, MOEA-CRL shows the best performance.

**Figure 14:**
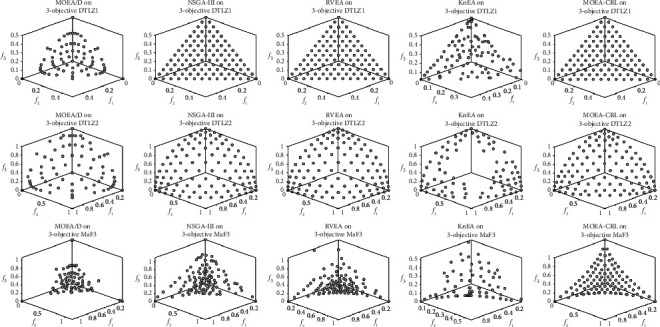
The nondominated solution set with the median IGD value among 30 runs obtained by MOEA/*D*, NSGA-III, RVEA, KnEA, and MOEA-CRL on DTLZ1, DTLZ2, and MaF3 with 3 objectives 4.

Figure 14 plots the nondominated solution sets for each algorithm that obtained on DTLZ1, DTLZ2, and MaF3 problems with three objectives, which are the mean values of the IGD after 30 runs. It can be further observed from Figure 14 that the MOEA-CRL obtains a uniformly distributed nondominated solution set on DTLZ1, DTLZ2, and MaF3. It can be seen that the proposed MOEA-CRL can not only perform well on the linear PF and concave PF, but also on the convex PF in MOPs. Specifically, most popular MOEAs perform well for the linear and concave PF such as DTLZ1 and DTLZ2, but the population diversity will deteriorate significantly on the test problems with convex PF and the Pareto solution sets lost the uniform distribution. As shown in Table 3 and Figure 14, it indicates that MOEA-CRL performs good convergence and diversity for MOPs, especially on the convex PF.

Table 4 lists the HV values gained by the MOEA-CRL and the four popular MOEAs on the test problems with 5 objectives and 10 objectives. Overall, MOEA-CRL achieved the best performance 16 times in a total of 38 experiments, while MOEA/D, NSGA-III, RVEA, and KnEA achieved the best performance 5, 6, 6, and 5 times, respectively. The evaluation results show that the overall performance of MOEA-CRL in dealing with MaOPs is better than the other four MOEAs, but the performance of MOEA-CRL deteriorates with the increase of dimension. For the regular Pareto test problems with 10 objectives, the advantage of MOEA-CRL is not significant. For the 9 irregular Pareto test problems, MOEA-CRL is competitive. Especially, MOEA-CRL performs better on the three concave PF test problems, MaF3, MaF11, and MaF15. Since the introduction of the nadir point will bring the performance gain on the convex PF more obvious, the cross-reference line method performs better on the convex PF than other multiobjective algorithms.

**Figure 15:**
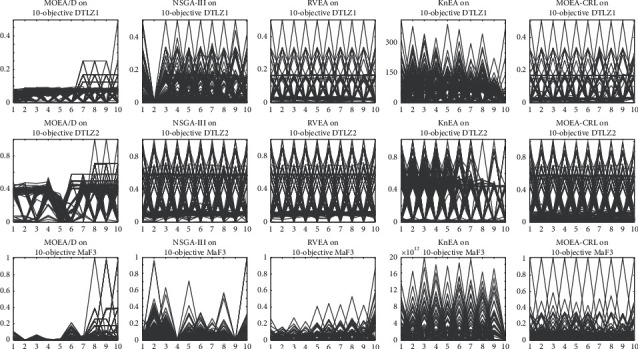
The nondominated solution set with the median HV value among 30 runs obtained by MOEA/D, NSGA-III, RVEA, KnEA, and MOEA-CRL on DTLZ1, DTLZ2, and MaF3 with 10 objectives.

Figure 15 plots the nondominated solution sets for each algorithm that obtained on DTLZ1, DTLZ2, and MaF3 with 10 objectives, which are the mean values of the HV after 30 runs. The parallel coordinate is a way of data visualization. Multiple vertical and parallel coordinate axes represent multiple dimensions, and the scale on the dimension represents the corresponding value on the objective. Each sample corresponds to a value in each dimension, and a connected polyline represents the sample. It can be further observed from Figure 15 that the MOEA-CRL obtains the uniformly distributed nondominated solution sets on DTLZ1, DTLZ2, and MaF3. The MOEA-CRL can still effectively enhance the population diversity. Specifically, MOEAs other than MOEA/D can maintain diversity and obtain the good uniformity of nondominated solution sets in test problems on DTLZ1 and DTLZ2 with the 10 objectives. However, the MOEA-CRL obtained a uniform distribution in test problem on MaF3 with the 10 objectives and the performance of other four MOEAs significantly deteriorates. Therefore, MOEA-CRL can maintain better population diversity on MaOPs as shown in Table 4 and Figure 15, especially on the convex PF. It should be noted that the convergence of MOEA-CRL deteriorates significantly as the dimension increases. The reason is that the nadir point reference lines are employed to enhance the diversity, which results in a decline of the convergence pressure.

Through the sensitivity analysis of the weight coefficient of the 3-objective problem, it can be concluded that the use of the nadir point does not weaken the convergence but increases the convergence pressure. However, as the objective dimension increases, the use of the nadir point will indeed cause the deterioration of convergence and even the failure to converge.

### 5.4. Sensitivity Analysis of Population Size

In the experiment, the population size N is set as same as the number of reference points since the reference points are generally associated with each candidate solution in most decomposition-based MOEAs. The number of reference points depends on the method of Das and Dennis [37].

The population size setting of MOEA-CRL proposed in this paper is flexible, and the number of reference points has less influence on it. The number of candidate solutions can be less than the number of reference points and also can be larger.

**Figure 16:**
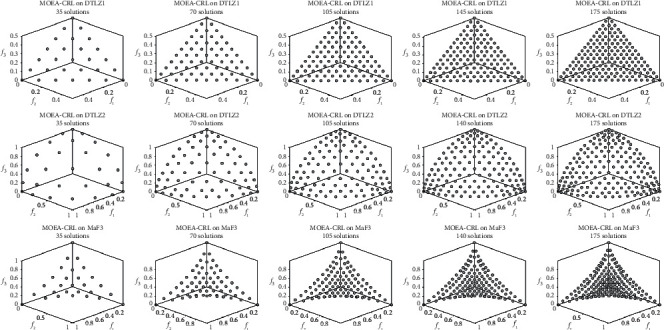
The nondominated solution sets of MOEA-CRL with population sizes of 35, 70, 105, 140, and 175 with 3 objectives.

MOEA-CRL with different population sizes was tested on DTLZ1, DTLZ2, and MaF3 with three objectives.  Figure 16 exhibits the nondominated solution sets of MOEA-CRL with population sizes of 35, 70, 105, 140, and 175, and the number of reference points is always set to 105. It can be shown that the Pareto solution sets obtained by MOEA-CRL are always uniformly distributed regardless of the population sizes. Therefore, MOEA-CRL provides greater flexibility for population size setting.

## 6. Conclusions and Remarks

In this paper, an evolutionary algorithm based on the adaptive cross-reference line method, called MOEA-CRL, is proposed to inherit the advantages of the ideal point reference line for better convergence and add the nadir point reference line for higher diversity. Especially, on the convex PF, MOEA-CRL solves the Pareto incompatibility problem and significantly enhances the population diversity. Furthermore, this paper proposed the DPD indicator based on the cross-reference lines. The properties of the ideal point reference line and the nadir point reference line are combined to solve the Pareto incompatibility problem as well as improve the performance of the MOEA-CRL on the convex PF. Based on the DPD evaluation strategy of the cross-reference line method, MOEA-CRL retains unique solution with the best convergence in each attraction region as a nondominated solution, which ensures that the Pareto solution set is distributed evenly. Finally, this paper proposed a cross-reference line adaptation method in order to enhance the performance of MOEA-CRL in dealing with the irregular problems.

The experimental results show the superiority of MOEA-CRL on the convex PF. It also has the competitiveness due to the adaptability of cross-reference lines while solving those MOPs and MaOPs with other types of PFs. Remarkably, the cross-reference line method is only used to calculate the DPD indicator. Therefore, the population size is irrelated to the number of the cross-reference lines, and subsequently, the population size setting is flexible. The proposed MOEA-CRL proves that the adaptive cross-reference line method is prospective for significantly improving the diversity especially in the convex PF.

In fact, the experimental results also clearly illustrate that the performance of MOEA-CRL deteriorates significantly with the increase of dimensions. That means that the cross-reference line method still poses the challenges in dealing with some research issues such as high-dimensional deterioration and more complex convex PF problems.

## Figures and Tables

**Algorithm 1:**
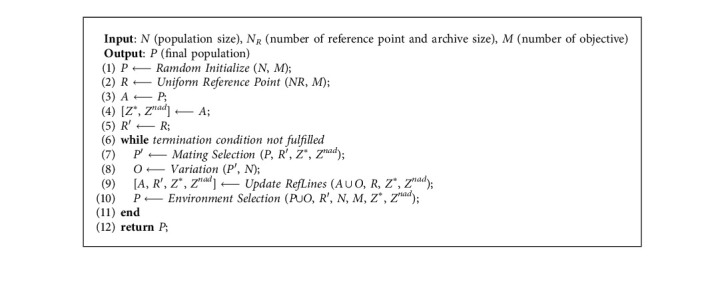
General framework of MOEA-CRL.

**Algorithm 2:**
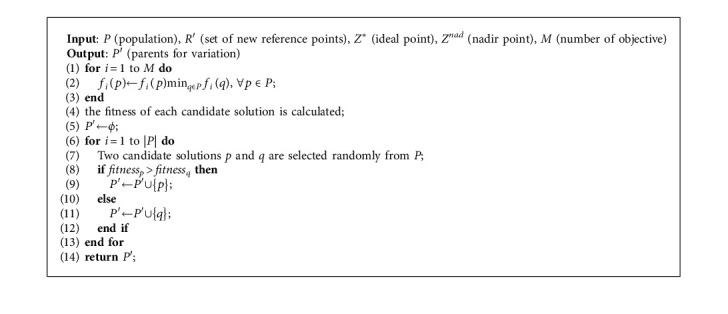
*Mating selection* (*P*, *R*′, *Z*^*∗*^, and *Z*^*nad*^).

**Algorithm 3:**
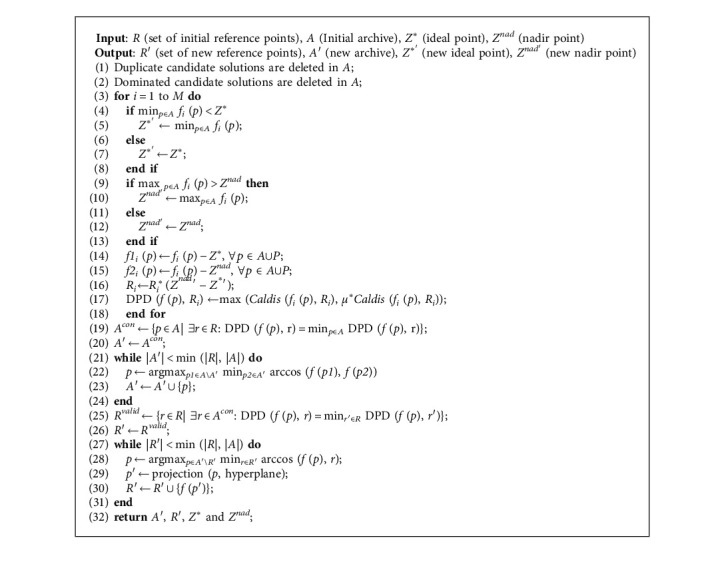
*Update RefLines* (*R*, *A*, *Z*^*∗*^, and *Z*^*nad*^).

**Algorithm 4:**
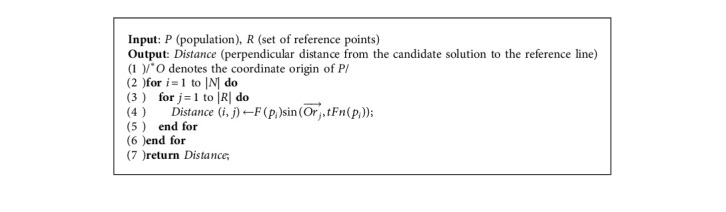
*Caldis* (*P* and *R*).

**Algorithm 5:**
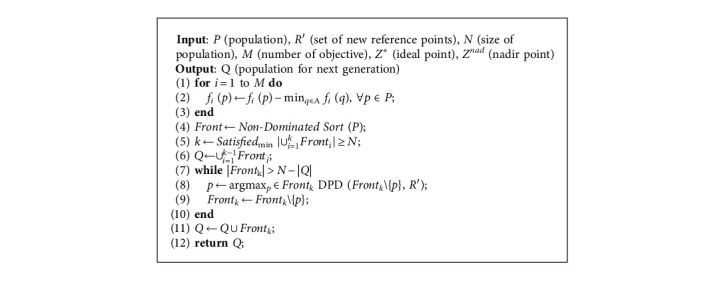
Environmental selection (*P*, *R*′, *N*, *M*, *Z*^∗^, and *Z*^*nad*^).

**Table 1:** Settings of the number of reference points for each number of objectives, where *p*1 and *p*2 denote the numbers of divisions on each objective for the boundary layer and the insider layer, respectively.

Number of objectives (*M*)	Parameter (*p*1, *p*2)	Number of reference points/population size (*N*)
3	13, 0	105
5	6, 0	210
10	3, 2	275

**Table 2:** Settings of the number of objectives, the number of decision variables, and the maximal number of generations for each test problem.

Test problem	*M*	*D*	Pareto front
Regular Pareto front			
DTLZ1	3, 5, 10	*M* −1 + 5	Linear
DTLZ3	3, 5, 10	*M* −1 + 10	Concave
DTLZ2, 4	3, 5, 10	*M* −1 + 10	Concave
WFG4-9	3, 5, 10	*M* −1 + 10	Concave
Irregular Pareto front			
DTLZ5-6	3, 5, 10	*M* −1 + 10	Mostly degenerate
DTLZ7	3, 5, 10	*M* −1 + 20	Disconnected
WFG1	3, 5, 10	*M* −1 + 10	Sharp tails
WFG2	3, 5, 10	*M* −1 + 10	Disconnected
WFG3	3, 5, 10	*M* −1 + 10	Mostly degenerate
MaF3	3, 5, 10	*M* −1 + 10	Convex
MaF11	3, 5, 10	*M* −1 + 10	Convex disconnected
MaF15	3, 5, 10	20^∗^M	Convex large scale

**Table 3:** Statistical results (mean values and standard deviations) of IGD value obtained by MOEA/D, NSGA-III, RVEA, KnEA, and MOEA-CRL on DTLZ1-DTLZ7, WFG1-WFG9, MaF3, MaF11, and MaF15 with 3 objectives.

Problem	*M*	*D*	MOEA/D	NSGA-III	RVEA	KnEA	MOEA-CRL
DTLZ1	3	7	2.8508*e* − 2 (4.30*e* − 6) −	1.8979*e* − 2 (4.28*e* − 6) ≈	1.8978*e* − 2 (5.63*e* − 6) ≈	5.0030*e* − 2 (2.51*e* − 2) −	1.8977*e* − 2 (2.29*e* − 5)
DTLZ2		12	6.9661*e* − 2 (5.68*e* − 5) −	5.0301*e* − 2 (4.52*e* − 7) −	5.0301*e* − 2 (4.21*e* − 7) −	6.6663*e* − 2 (2.44*e* − 3) −	4.6814*e* − 2 (5.07*e* − 5)
DTLZ3		12	1.0106*e* − 1 (1.20*e* − 1) −	5.0394*e* − 2 (1.67*e* − 4) −	5.0355*e* − 2 (7.33*e* − 5) −	1.0817*e* − 1 (2.89*e* − 2) −	4.7075*e* − 2 (1.21*e* − 4)
DTLZ4		12	2.3255*e* − 1 (3.36*e* − 1) −	1.3215*e* − 1 (1.86*e* − 1) ＋	5.0300*e* − 2 (4.47*e* − 7) ≈	1.5318*e* − 1 (2.69*e* − 1) ＋	2.2800*e* − 1 (2.42*e* − 1)
WFG4	3	12	2.7142*e* − 1 (6.38*e* − 4) −	2.0405*e* − 1 (3.67*e* − 5) −	2.0800*e* − 1 (2.21*e* − 3) −	2.4949*e* − 1 (7.34*e* − 3) −	1.9326*e* − 1 (4.61*e* − 4)
WFG5			2.8760*e* − 1 (9.10*e* − 4) −	2.1444*e* − 1 (3.75*e* − 5) −	2.1580*e* − 1 (6.17*e* − 4) −	2.6120*e* − 1 (1.07*e* − 2) −	2.0682*e* − 1 (2.34*e* − 4)
WFG6			2.9642*e* − 1 (1.27*e* − 2) −	2.1871*e* − 1 (8.11*e* − 3) ≈	2.2604*e* − 1 (1.00*e* − 2) −	2.8667*e* − 1 (1.28*e* − 2) −	2.1759*e* − 1 (1.29*e* − 2)
WFG7			2.7206*e* − 1 (6.18*e* − 4) −	2.0414*e* − 1 (5.96*e* − 5) −	2.0612*e* − 1 (9.83*e* − 4) −	2.4178*e* − 1 (9.90*e* − 3) −	1.9289*e* − 1 (3.86*e* − 4)
WFG8			3.1847*e* − 1 (6.38*e* − 3) −	2.6527*e* − 1 (2.75*e* − 3) −	2.8098*e* − 1 (5.23*e* − 3) −	3.3436*e* − 1 (7.90*e* − 3) −	2.6066*e* − 1 (2.48*e* − 3)
WFG9			2.7920*e* − 1 (3.28*e* − 2) −	2.0537*e* − 1 (7.59*e* − 4) −	2.0722*e* − 1 (1.72*e* − 3) −	2.2643*e* − 1 (6.75*e* − 3) −	1.9470*e* − 1 (8.91*e* − 4)
＋/−/≈			0/10/0	1/8/1	1/8/1	1/9/0	
DTLZ5	3	12	1.2417*e* − 2 (1.51*e* − 6) −	1.1730*e* − 2 (1.24*e* − 3) −	5.8469*e* − 2 (1.08*e* − 3) −	1.0462*e* − 2 (2.65*e* − 3) −	5.7501*e* − 3 (3.73*e* − 4)
DTLZ6		12	1.2419*e* − 2 (7.06*e* − 7) −	1.7437*e* − 2 (2.69*e* − 3) −	5.9099*e* − 2 (3.01*e* − 3) −	4.7828*e* − 3 (3.60*e* − 4) −	4.2868*e* − 3 (3.45*e* − 5)
DTLZ7		22	2.4700*e* − 1 (8.06*e* − 2) −	7.0580*e* − 2 (2.35*e* − 3) ＋	1.0489*e* − 1 (3.58*e* − 3) ＋	8.4509*e* − 2 (7.48*e* − 2) ＋	2.0368*e* − 1 (1.79*e* − 1)
WFG1	3	12	2.1051*e* − 1 (1.22*e* − 2) −	1.3620*e* − 1 (2.39*e* − 3) ＋	1.5393*e* − 1 (6.17*e* − 3) −	1.8632*e* − 1 (7.92*e* − 3) −	1.4766*e* − 1 (2.95*e* − 3)
WFG2		12	2.1674*e* − 1 (1.53*e* − 3) −	1.5105*e* − 1 (1.13*e* − 3) ≈	1.6491*e* − 1 (5.59*e* − 3) −	1.8398*e* − 1 (8.28*e* − 3) −	1.5058*e* − 1 (9.94*e* − 4)
WFG3		12	4.1686*e* − 2 (3.12*e* − 4) ＋	8.8550*e* − 2 (6.34*e* − 3) ＋	2.1668*e* − 1 (8.51*e* − 3) −	9.6049*e* − 2 (8.33*e* − 3) −	8.9822*e* − 2 (8.87*e* − 3)
Convex							
MaF3	3	12	1.5083*e* − 1 (9.68*e* − 2) −	4.3564*e* − 2 (3.22*e* − 4) −	3.8402*e* − 2 (4.17*e* − 4) −	1.4269*e* − 1 (6.04*e* − 2) −	3.4876*e* − 2 (8.30*e* − 4)
MaF11		12	2.1673*e* − 1 (1.60*e* − 3) −	1.5147*e* − 1 (9.71*e* − 4) ≈	1.6463*e* − 1 (4.37*e* − 3) −	1.8721*e* − 1 (9.46*e* − 3) −	1.5082*e* − 1 (1.11*e* − 3)
MaF15		5	4.5845*e* − 1 (2.10*e* − 1) −	3.6301*e* − 1 (1.82*e* − 1) −	9.0510*e* − 1 (2.23*e* − 1) −	5.1952*e* − 1 (1.64*e* − 1) −	2.6890*e* − 1 (3.57*e* − 2)
＋/−/≈			1/8/0	3/4/2	1/8/0	1/8/0	

**Table 4:** Statistical results (mean values and standard deviations) of HV value obtained by MOEA/D, NSGA-III, RVEA, KnEA, and MOEA-CRL on DTLZ1-DTLZ7, WFG1-WFG9, MaF3, MaF11, and MaF15 with 5 objectives and 10 objectives.

Problem	*M*	MOEA/D	NSGA-III	RVEA	KnEA	MOEA-CRL
DTLZ1	5	9.0873*e* − 1 (8.52*e* − 2) −	9.7979*e* − 1 (1.56*e* − 4) ≈	9.7984*e* − 1 (1.55*e* − 4) ≈	6.2616*e* − 1 (1.64*e* − 1) −	9.7988*e* − 1 (1.63*e* − 4)
	10	9.7273*e* − 1 (5.32*e* − 3) −	9.8648*e* − 1 (3.26*e* − 2) −	9.9967*e* − 1 (1.88*e* − 5) ≈	0.0000*e* + 0 (0.00*e* + 0) −	9.9973*e* − 1 (1.10*e* − 4)

DTLZ2	5	7.1112*e* − 1 (5.08*e* − 4) −	8.1269*e* − 1 (4.52*e* − 4) −	8.1252*e* − 1 (4.48*e* − 4) −	7.9064*e* − 1 (3.54*e* − 3) −	8.1626*e* − 1 (9.62*e* − 4)
	10	6.2665*e* − 1 (2.70*e* − 2) −	9.4539*e* − 1 (3.51*e* − 2) −	9.6963*e* − 1 (1.72*e* − 4) −	9.5650*e* − 1 (4.53*e* − 3) −	9.7102*e* − 1 (1.70*e* − 3)

DTLZ3	5	4.3566*e* − 1 (1.27*e* − 1) −	7.7543*e* − 1 (2.88*e* − 3) ＋	7.7757*e* − 1 (1.36*e* − 3) ＋	3.9573*e* − 1 (1.31*e* − 1) −	7.3975*e* − 1 (3.36*e* − 3)
	10	6.4046*e* − 1 (1.97*e* − 2) −	3.3590*e* − 1 (4.26*e* − 1) −	9.6443*e* − 1 (9.40*e* − 3) ＋	0.0000*e* + 0 (0.00*e* + 0) −	9.6234*e* − 1 (2.68*e* − 1)

DTLZ4	5	2.7690*e* − 1 (1.65*e* − 1) −	7.3262*e* − 1 (6.50*e* − 2) ＋	7.7683*e* − 1 (1.68*e* − 2) ＋	7.6308*e* − 1 (4.31*e* − 3) ＋	7.2000*e* − 1 (5.17*e* − 2)
	10	6.2169*e* − 1 (2.11*e* − 2) −	9.6721*e* − 1 (1.24*e* − 2) −	9.6984*e* − 1 (1.99*e* − 4) −	9.5637*e* − 1 (4.37*e* − 3) −	9.7165*e* − 1 (1.51*e* − 3)

WFG4	5	6.2968*e* − 1 (8.71*e* − 4) −	8.0469*e* − 1 (8.35*e* − 4) −	8.0565*e* − 1 (1.10*e* − 3) ≈	7.8730*e* − 1 (2.17*e* − 3) −	8.0575*e* − 1 (1.74*e* − 3)
	10	5.8107*e* − 1 (5.61*e* − 2) −	9.4578*e* − 1 (3.92*e* − 3) ＋	9.4326*e* − 1 (3.57*e* − 3) ＋	9.5750*e* − 1 (1.56*e* − 3) ＋	9.1518*e* − 1 (5.08*e* − 3)

WFG5	5	5.9294*e* − 1 (1.98*e* − 2) −	7.6126*e* − 1 (3.68*e* − 4) ＋	7.6092*e* − 1 (4.04*e* − 4) ＋	7.4495*e* − 1 (2.82*e* − 3) ＋	7.3465*e* − 1 (2.49*e* − 3)
	10	5.3478*e* − 1 (1.75*e* − 2) −	8.9907*e* − 1 (6.43*e* − 4) ＋	8.9790*e* − 1 (1.17*e* − 3) ＋	8.9664*e* − 1 (8.14*e* − 4) ＋	8.4566*e* − 1 (3.56*e* − 3)

WFG6	5	5.5065*e* − 1 (3.13*e* − 2) −	7.4242*e* − 1 (1.58*e* − 2) ＋	7.4662*e* − 1 (1.44*e* − 2) ＋	7.2041*e* − 1 (1.03*e* − 2) ≈	7.2008*e* − 1 (1.20*e* − 2)
	10	4.7917*e* − 1 (9.01*e* − 2) −	8.6935*e* − 1 (1.66*e* − 2) ≈	8.6291*e* − 1 (2.01*e* − 2) ≈	8.6854*e* − 1 (1.59*e* − 2) ≈	8.7014*e* − 1 (1.72*e* − 2)

WFG7	5	6.2952*e* − 1 (1.76*e* − 3) −	8.0771*e* − 1 (6.15*e* − 4) ＋	8.0685*e* − 1 (6.00*e* − 4) ＋	7.9537*e* − 1 (2.41*e* − 3) ＋	7.9284*e* − 1 (1.74*e* − 3)
	10	6.1632*e* − 1 (3.69*e* − 2) −	9.4638*e* − 1 (2.02*e* − 2) ＋	9.4552*e* − 1 (2.93*e* − 3) −	9.5737*e* − 1 (6.09*e* − 3) ＋	9.4268*e* − 1 (2.70*e* − 3)

WFG8	5	3.2668*e* − 1 (1.10*e* − 2) −	6.9440*e* − 1 (3.44*e* − 3) −	6.9749*e* − 1 (1.55*e* − 3) ≈	6.6088*e* − 1 (4.04*e* − 3) −	6.9786*e* − 1 (2.26*e* − 3)
	10	5.2900*e* − 1 (2.26*e* − 2) −	8.3115*e* − 1 (2.65*e* − 2) −	7.4198*e* − 1 (7.45*e* − 2) −	8.1031*e* − 1 (5.88*e* − 2) −	8.5967*e* − 1 (2.09*e* − 2)

WFG9	5	4.1801*e* − 1 (7.01*e* − 2) −	7.6559*e* − 1 (4.98*e* − 3) ＋	7.6952*e* − 1 (3.01*e* − 3) ＋	7.6796*e* − 1 (3.00*e* − 3) ＋	7.4665*e* − 1 (4.89*e* − 3)
	10	5.3897*e* − 1 (5.22*e* − 2) －	8.6420*e* − 1 (4.42*e* − 2) ＋	8.7342*e* − 1 (1.19*e* − 2) ＋	9.0581*e* − 1 (3.21*e* − 2) ＋	8.3182*e* − 1 (1.92*e* − 2)
＋/−/≈		0/20/0	10/8/2	10/5/5	8/10/2	

DTLZ5	5	1.0968*e* − 1 (8.30*e* − 3) ≈	1.0483*e* − 1 (1.62*e* − 2) ≈	9.1890*e* − 2 (1.22*e* − 3) −	7.1580*e* − 2 (2.93*e* − 2) −	1.0808*e* − 1 (5.72*e* − 3)
	10	9.7922*e* − 2 (3.20*e* − 4) ＋	2.8809*e* − 2 (3.32*e* − 2) −	9.0906*e* − 2 (1.34*e* − 4) ＋	3.5304*e* − 2 (3.14*e* − 2) −	8.2365*e* − 2 (2.16*e* − 2)

DTLZ6	5	9.4634*e* − 2 (6.13*e* − 3) ≈	5.7651*e* − 2 (4.09*e* − 2) −	9.9670*e* − 2 (4.73*e* − 3) ≈	9.1029*e* − 2 (2.57*e* − 3) −	9.2650*e* − 2 (2.20*e* − 2)
	10	9.8124*e* − 2 (2.62*e* − 4) ＋	3.0230*e* − 3 (1.66*e* − 2) −	9.2019*e* − 2 (9.23*e* − 4) ＋	0.0000*e* + 0 (0.00*e* + 0) −	9.1491*e* − 2 (3.74*e* − 2)

DTLZ7	5	1.7168*e* − 1 (5.33*e* − 2) −	2.3157*e* − 1 (9.31*e* − 3) ＋	2.1343*e* − 1 (8.52*e* − 3) −	2.4843*e* − 1 (1.07*e* − 2) ＋	2.2732*e* − 1 (3.03*e* − 3)
	10	4.5806*e* − 3 (7.31*e* − 3) −	1.7116*e* − 1 (7.07*e* − 3) ＋	1.3371*e* − 1 (2.42*e* − 2) ＋	9.1243*e* − 2 (2.89*e* − 2) ＋	7.4082*e* − 2 (2.95*e* − 2)

WFG1	5	9.4334*e* − 1 (7.14*e* − 2) −	9.7370*e* − 1 (2.11*e* − 2) −	9.8276*e* − 1 (2.89*e* − 2) ≈	9.9246*e* − 1 (1.41*e* − 3) −	9.9835*e* − 1 (1.60*e* − 2)
	10	5.0234*e* − 1 (1.69*e* − 1) −	9.4008*e* − 1 (4.39*e* − 2) ＋	9.9015*e* − 1 (2.42*e* − 2) ＋	9.9756*e* − 1 (8.35*e* − 4) ＋	7.4040*e* − 1 (5.17*e* − 2)

WFG2	5	9.6566*e* − 1 (3.42*e* − 2) −	9.9555*e* − 1 (8.56*e* − 4) −	9.9404*e* − 1 (1.15*e* − 3) −	9.9320*e* − 1 (7.41*e* − 4) −	9.9683*e* − 1 (7.33*e* − 4)
	10	9.9648*e* − 1 (2.10*e* − 3) ＋	9.9700*e* − 1 (1.46*e* − 3) ＋	9.8471*e* − 1 (4.33*e* − 3) −	9.9306*e* − 1 (1.47*e* − 3) ＋	9.9191*e* − 1 (2.27*e* − 3)

WFG3	5	9.1998*e* − 2 (3.47*e* − 4) −	1.6765*e* − 1 (1.32*e* − 2) ≈	1.5967*e* − 1 (1.56*e* − 2) ≈	7.7484*e* − 2 (1.82*e* − 2) −	1.6083*e* − 1 (1.27*e* − 2)
	10	7.5376*e* − 2 (7.87*e* − 3) ＋	2.4516*e* − 4 (1.34*e* − 3) −	0.0000*e* + 0 (0.00*e* + 0) −	0.0000*e* + 0 (0.00*e* + 0) −	4.0606*e* − 2 (7.87*e* − 3)

MaF3	5	9.9646*e* − 1 (1.07*e* − 4) −	9.9870*e* − 1 (2.01*e* − 3) −	9.9895*e* − 1 (4.31*e* − 4) −	8.8686*e* − 1 (1.27*e* − 1) −	9.9975*e* − 1 (7.14*e* − 4)
	10	9.9993*e* − 1 (4.54*e* − 5) ≈	2.9125*e* − 1 (4.53*e* − 1) −	9.8343*e* − 1 (5.59*e* − 2) −	0.0000*e* + 0 (0.00*e* + 0) −	9.9997*e* − 1 (1.30*e* − 1)

MaF11	5	9.7508*e* − 1 (2.07*e* − 2) −	9.9583*e* − 1 (5.38*e* − 4) −	9.9397*e* − 1 (1.11*e* − 3) −	9.9299*e* − 1 (8.84*e* − 4) −	9.9751*e* − 1 (5.92*e* − 4)
	10	9.3320*e* − 1 (6.36*e* − 2) −	9.9641*e* − 1 (2.18*e* − 3) −	9.8256*e* − 1 (4.48*e* − 3) −	9.9284*e* − 1 (8.14*e* − 4) −	9.9818*e* − 1 (2.03*e* − 3)

MaF15	5	2.9995*e* − 2 (1.11*e* − 2) ＋	0.0000*e* + 0 (0.00*e* + 0) −	2.4759*e* − 2 (1.06*e* − 2) ＋	0.0000*e* + 0 (0.00*e* + 0) −	1.0571*e* − 2 (5.05*e* − 3)
	10	3.0314*e* − 11 (1.16*e* − 10) −	0.0000*e* + 0 (0.00*e* + 0) −	1.9696*e* − 7 (2.48*e* − 7) −	0.0000*e* + 0 (0.00*e* + 0) −	2.6803*e* − 7 (2.42*e* − 10)
＋/−/≈		5/10/3	4/12/2	5/10/3	4/14/0	

## Data Availability

The data used to support the findings of this study are available from the corresponding author upon request.
